# Novel Biallelic Variants and Phenotypic Features in Patients with *SLC38A8*-Related Foveal Hypoplasia

**DOI:** 10.3390/ijms22031130

**Published:** 2021-01-24

**Authors:** Elena R. Schiff, Vijay K. Tailor, Hwei Wuen Chan, Maria Theodorou, Andrew R. Webster, Mariya Moosajee

**Affiliations:** 1 Moorfields Eye Hospital NHS Foundation Trust, London EC1V 2PD, UK; e.schiff@ucl.ac.uk (E.R.S.); vijay.tailor.09@ucl.ac.uk (V.K.T.); hwei_wuen_chan@nuhs.edu.sg (H.W.C.); mtheodorou@nhs.net (M.T.); Andrew.webster@ucl.ac.uk (A.R.W.); 2 Institute of Ophthalmology, University College London, London EC1V 9EL, UK; 3 Department of Experimental Psychology, University College London, London WC1H 0AP, UK; 4 Department of Ophthalmology, National University Hospital, Singapore S118177, Singapore; 5 Great Ormond Street Hospital for Children NHS Foundation Trust, London WC1N 3JH, UK; 6 The Francis Crick Institute, London NW1 1AT, UK

**Keywords:** foveal hypoplasia, *SLC38A8*, nystagmus, chiasmal misrouting, anterior segment dysgenesis

## Abstract

Biallelic pathogenic variants in solute carrier family 38 member 8, *SLC38A8*, cause a pan-ocular autosomal recessive condition known as foveal hypoplasia 2, FVH2, characterised by foveal hypoplasia, nystagmus and optic nerve chiasmal misrouting. Patients are often clinically diagnosed with ocular albinism, but foveal hypoplasia can occur in several other ocular disorders. Here we describe nine patients from seven families who had molecularly confirmed biallelic recessive variants in *SLC38A8* identified through whole genome sequencing or targeted gene panel testing. We identified four novel sequence variants (p.(Tyr88*), p.(Trp145*), p.(Glu233Gly) and c.632+1G>A). All patients presented with foveal hypoplasia, nystagmus and reduced visual acuity; however, one patient did not exhibit any signs of chiasmal misrouting, and three patients had features of anterior segment dysgenesis. We highlight these findings in the context of 30 other families reported to date. This study reinforces the importance of obtaining a molecular diagnosis in patients whose phenotype overlap with other inherited ocular conditions, in order to support genetic counselling, clinical prognosis and family planning. We expand the spectrum of *SLC38A8* mutations which will be relevant for treatment through future genetic-based therapies.

## 1. Introduction

The human fovea is located at the most central part of the macula, a 1.5 mm wide area most densely packed with cone photoreceptors. Its anatomy is distinct from other retinal regions, having a single layer of cones in the neurosensory retina with the inner nuclear and ganglion cell layer displaced radially outwards forming the foveal pit. Foveal hypoplasia (FVH) is the absence of this normal foveal depression, and the resultant continuation of all retinal layers and retinal vasculature through the foveal region. Grading systems describe the extent of FVH [1,2], and this can occur in isolation or as a feature of several ocular disorders including albinism, aniridia, retinopathy of prematurity, and microphthalmia. More recent associations, identified through high-resolution spectral domain optical coherence tomography (OCT) imaging, are achromatopsia, optic nerve hypoplasia, familial exudative vitreoretinopathy and Stickler syndrome [3]. For most—but not all—of these conditions, FVH is accompanied by nystagmus and decreased visual acuity, but this is dependent on the grade and severity.

When FVH occurs with optic nerve misrouting, it is usually an indication of albinism. Recessive mutations in the *SLC38A8* (solute carrier family 38 member 8) gene on chromosome 16q23.3 have been shown, however, to cause FVH and optic nerve misrouting independent of the pigmentation defects associated with oculocutaneous albinism or ocular albinism [4,5]. Additionally known as foveal hypoplasia 2 with or without optic nerve misrouting and/or anterior segment dysgenesis (FVH2, OMIM #609218), *SLC38A8* is a pan-ocular autosomal recessive condition, additionally labelled with the acronym FHONDA (foveal hypoplasia, optic nerve decussation and anterior segment dysgenesis without albinism) [6]. 

*SLC38A8* has 10 exons and encodes an amino acid transporter protein, which is one of 11 members of the SLC38 sodium-coupled neutral amino acid transporter (SNAT) family. The 435-amino acid protein SNAT8, has eleven transmembrane domains, an extracellular N-terminus and an intracellular C-terminal tail. The functional characterisation of SNAT8 showed that it is ubiquitously expressed in neurons and has a broad substrate profile with high preference for transporting l-glutamine, l-alanine, l-arginine, l-histidine and l-aspartate. As a glutamine transporter, a key role for regulating levels of glutamate and gamma aminobutyric acid (GABA) in the brain has been suggested [7]. GABA/GABAergic dysregulation has a significant impact on brain function from developmental to neurodegenerative changes as seen in Alzheimer’s disease. It remains unknown whether this disruption in early development impacts on axonal projection, hence contributing towards chiasmal misrouting. Using human eye tissues, *SLC38A8* was shown to be located throughout the neural retina, specifically in the inner and outer plexiform layers and the photoreceptor layer [4]. 

To date, 33 unique disease-causing mutations have been reported in 30 families of multiple ethnicities (Summarised in Table S1) [4,5,8,9,10,11,12], with an additional four only reported in ClinVar (total = 37). Almost half (*n* = 16) are missense mutations with the remainder being frameshift (*n* = 6), nonsense (*n* = 4), splice variant (*n* = 4) and deletion mutations (*n* = 7). In this study we detail the clinical and molecular findings in seven families with biallelic *SLC38A8* mutations causing foveal hypoplasia, identified through whole genome sequencing (WGS), and through targeted gene panel testing, thus expanding the phenotypic and genotypic spectrum of *SLC38A8*-oculopathy.

## 2. Results

### 2.1. Molecular Variant Findings

**Figure 1:**
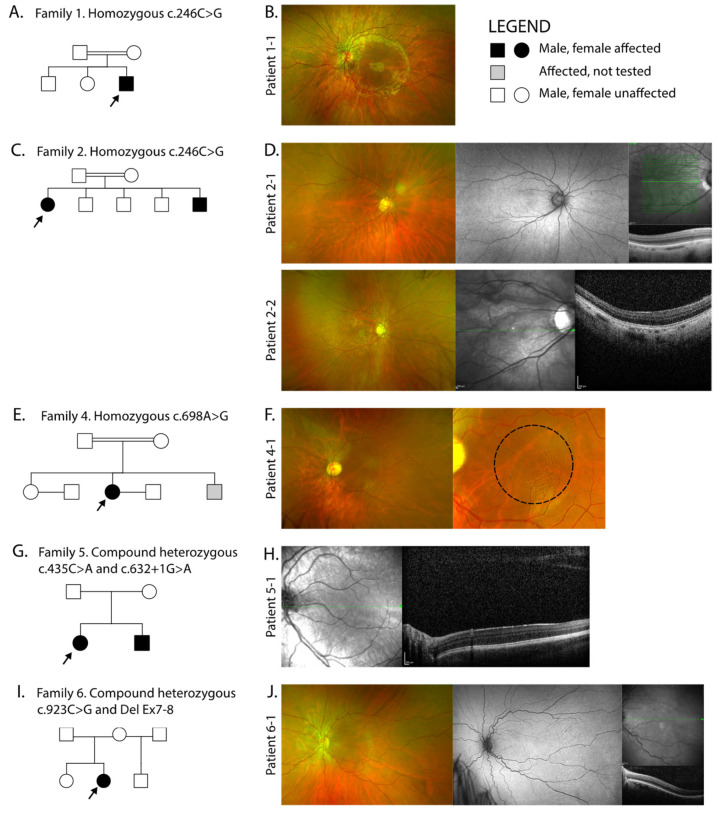
Pedigrees of families with molecularly confirmed *SLC38A8* biallelic variants, and corresponding retinal imaging. (**A**) Family 1 are consanguineous and (**B**) proband 1-1 with left ultra-widefield (UWF) colour fundus imaging; (**C**) Family 2 are consanguineous and (**D**) shows proband 2-1 right UWF colour fundus image, corresponding UWF fundus autofluorescence (FAF) with no defined fovea (usually depicted by a hypoautoautofluorescent spot in healthy individuals) and spectral-domain OCT (SD-OCT) demonstrating foveal hypoplasia through the centre of the right macula. Sibling 2-2 right UWF colour fundus and SD-OCT with foveal hypoplasia; (**E**) Family 4 are consanguineous and (**F**) proband 4-1 left UWF colour fundus with close up of the macula showing the concentric ring sign within the black dashed circle, which is seen in patients with foveal hypoplasia; (**G**) Family 5 and (**H**) proband 5-1 showing left macula SD-OCT with foveal hypoplasia; (**I**) Family 6 and (**J**) proband 6-1 with left UWF colour fundus, UWF FAF with no foveal definition and SD-OCT with foveal hypoplasia. Imaging was not available in family 3 and 7.

**Figure 2:**
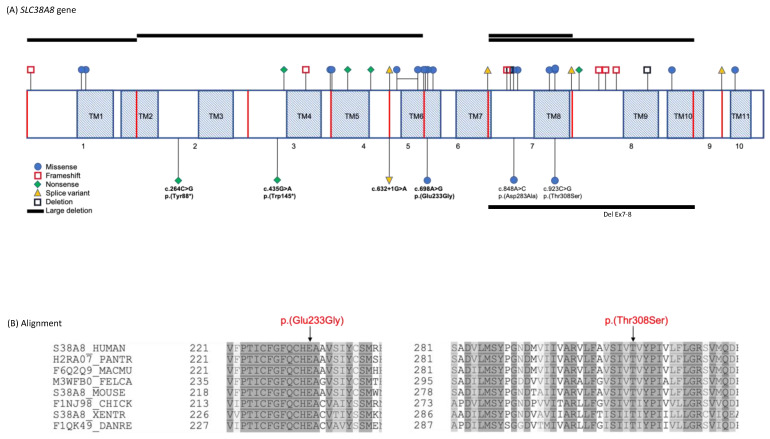
*SLC38A8* variants and corresponding protein alignment of two missense mutations. (**A**) Schematic representation of *SLC38A8* gene transcript showing localisation of mutations reported to date (Table S1) above the transcript and mutations described in this study below, novel mutations in bold. Red vertical lines represent exon boundaries; shaded areas represent the eleven transmembrane domains, TM1-TM11; (**B**) Multiple organism sequence alignment of regions spanning the p.Glu233Gly and p.Thr308Ser amino acids of the *SLC38A8* protein, showing their strong evolutionary conservation (highly conserved in darker grey moderately conserved in lighter grey). Alignment was performed using https://www.uniprot.org/align/. Protein sequences used for alignment are S38A8 in the human, H2RA07 in the chimpanzee, F6Q2Q9 in the rhesus monkey, M3WFB0 in the cat, S38A8 in the mouse, F1NJ98 in the chicken, S38A8 in the frog and F1QK49 in the zebrafish.

Nine patients (6 females and 3 males) from 7 families with FVH and nystagmus were recruited into this study. Four families were of South Asian ethnicity with reported consanguinity, and three were Caucasian and non-consanguineous. The pedigrees of each family are shown in Figure 1 and the molecular findings summarised in Table 1. We identified 4 novel variants in *SLC38A8* accounting for families 1–5; two nonsense (c.264C>G p.[Tyr88*] and c.435G>A p.[Trp145*]), one missense (c.698A>G, p.[Glu233Gly]) and one splice site (c.632+1G>A). A known missense heterozygous variant was found in family 6 (c.923C>G, p.[Thr308Ser]) together with a heterozygous deletion of exons 7 and 8 and a known homozygous missense was found in family 7 (c.848A>C, p.[Asp283Ala]). There are 37 reported mutations to date, and along with our novel variants these span all 10 exons (Figure 2A). The genotype of families 1, 3, 4 and 5 were reported in our previous study reporting the genetic outcomes of a large cohort of non-retinal developmental eye disease patients recruited into the 100,000 Genomes Project, but no detailed phenotype data was provided [13]. Variants were shown to be in trans in five of seven families where parents, all unaffected, were available for segregation analysis. Probands from families three and four were analysed as singletons. 

The nonsense mutation c.264C>G, p.(Tyr88*), observed homozygously in families 1, 2 and 3, lies between transmembrane domains 2–3, in a region deplete of reported variants (Figure 2A). It is likely that transcripts encoding nonsense and frameshift variants will be removed by nonsense-mediated decay or alternatively result in a truncated protein. In family 5, the novel nonsense mutation c.435G>A, p.(Trp145*) lies between transmembrane domains 3-4, in proximity to another nonsense mutation, p.(Gln149Ter), listed in ClinVar (rs14689932). This may be a region susceptible to protein function disruption. The novel splice variant c.632+1G>A observed in trans with p.(Trp145*) in family 5 alters the conserved canonical donor splice site at the exon4-intron4 junction, and a different splice mutation at this site, c.632+2T>G, was recently described [12]. The novel missense c.698A>G, p.(Glu233Gly) in family 4 is predicted to be probably damaging/damaging/disease-causing by Polyphen2, SIFT and Mutation Taster, respectively. It involves a highly conserved residue within a conserved block (Figure 2B) and is absent from gnomAD. It occurs in the sixth transmembrane domain which harbours 5 other missense variants and is the most frequently affected domain. The previously reported [9] missense c.923C>G, p.(Thr308Ser) heterozygous variant in family 6 is similarly predicted to be likely pathogenic and is also positioned in a highly conserved amino acid residue block (Figure 2B). It is also absent from gnomAD, though a different amino acid missense at this position, p.(Thr308Ala), has also been reported [9]. It is the only mutated amino acid in the eighth transmembrane domain. It is therefore likely that these missense mutations occur in locations that are critical for the normal function of the protein. A deletion spanning exons 7 and 8 was found in trans with c.923C>G, p.(Thr308Ser) in family 6. An exon 7-8 deletion has also been described in one patient of French origin in trans with c.697G>A, p.(Glu233Lys) (see Table S1).

The homozygous missense variant c.848A>C, p.(Asp283Ala) found in family 7 has previously been reported in both a homozygous [8,14] and compound heterozygous state with the pathogenic variant c.527C>G, p.(Thr176Arg) [9]. It occurs in the extracellular domain between the 7th and 8th transmembrane domains and structural modelling predicted a destabilizing effect on the protein by altering the electrostatic potential within the channel pore [8]. Family 7 were of Ashkenazi Jewish descent and this variant is relatively common in this population (0.006178 compared to 0.0002653 in gnomAD overall).

### 2.2. Clinical Findings

The ophthalmic and ocular findings of patients with *SLC38A8* variants are summarised in Table 2 and Figure 1. The mean ± SD BCVA was 0.73 ± 0.15 LogMAR. All nine patients had FVH and eight out of nine patients demonstrated a horizontal nystagmus waveform type (7 with pendular nystagmus and 1 had a jerk with extended foveation), and one patient had a rotary nystagmus. FVH was recorded through either ophthalmic examination or retinal imaging using fundus autofluorescence (FAF) or SD-OCT. FAF revealed an absence of the foveal hypoautofluorescent spot, hence no defined fovea as shown in Figure 1D and J. SD-OCT was challenging due to lack of patient fixation resulting from the FVH and nystagmus, however using the best single line scans, grade 4 FVH was seen in six families, graded according to Thomas et al. [8] Grade 4 FVH includes absence of the following features: (i) plexiform layers, (ii) foveal pit, (iii) outer segment lengthening and (iv) outer nuclear layer widening (Figure 1D,F,H,J). Close inspection of the UWF colour fundus images revealed the concentric macular ring sign in four patients; 1-1, 2-1, 4-1 (shown in Figure 1F) and 6-1. Its appearance is reliant on the quality of the images, which as with OCT is confounded by age and fixation. This is a documented sign of FVH which has been postulated to arise from the radially symmetric orientation of the axon photoreceptors (Henle fibre layer) and possibly also the retinal nerve fibre layer surrounding the fovea [15,16].

Seven patients had a strabismus with exotropia in five patients and esotropia in two. Six patients who underwent electrophysiology with pattern appearance visual evoked potentials (VEP) had evidence of intracranial misrouting with contralateral predominance (crossed asymmetry). Three patients (2-2, 4-1 and 6-1) had variable degrees of anterior segment dysgenesis, two had bilateral posterior embryotoxon, 4-1 also had bilateral iris adhesions to the cornea and bilateral blue dot cataracts, and 6-1 had bilateral shallow anterior chamber. 

In family 2, of Indian Gujarati background, both the affected siblings were initially reported as having paler skin than their three unaffected siblings and parents, however, over time their skin pigmentation was similar to that of the rest of the family. Their hair and eyes were no different to that of their parents. In family 5, of Caucasian ethnicity, it was noted that the two affected children had fair hair and complexion, but this was in common with both their parents and many fair-haired and blue-eyed relatives. The remaining patients had normal hair and skin pigment compared to relatives and no ocular hypopigmentation.

**Figure 3:**
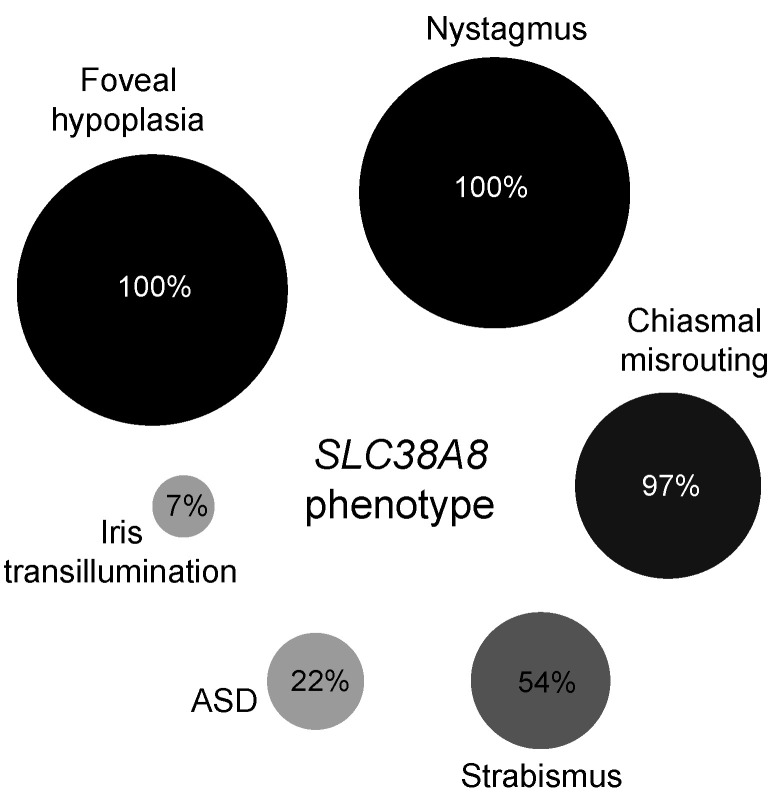
Bubble plot representing the relative frequency of phenotypic features in 58 reported cases (including our cohort) in 37 families. Anterior segment dysgenesis encompasses all recorded features including posterior embryotoxon, Axenfeld anomaly, blue-dot cataract, shallow anterior segments and iris transillumination.

All previously documented cases in the literature (30 families) were analysed for the prevalence of *SLC38A8* phenotypic features including our cohort (seven families). Of the 58 affected individuals in 37 families, 100% (*n* = 58) had foveal hypoplasia and nystagmus, while 97% (29 out of the 30 who underwent VEP testing) showed chiasmal misrouting. Anterior segment dysgenesis was documented in 22% (13/58) of patients with 21% (*n* = 12) having posterior embryotoxon and 5% (*n* = 3) showing Axenfeld anomaly (defined as posterior embryotoxon with peripheral anterior iris adhesions). Strabismus was observed in 54% (20/37) and iris transillumination in 7% (4/58) (see Figure 3 and Table S2).

## 3. Discussion

This study reports four novel variants in *SLC38A8* amongst 7 families, with the associated clinical features, and compares these to previously described molecularly confirmed cases. The homozygous nonsense mutation c.264C>G, p.(Tyr88*) was found in three unrelated families from different regions of South Asia. This variant was rare in gnomAD, but found with an allele frequency of 0.0002614 only in the South Asian population suggesting that this represents a possible founder mutation.

Foveal hypoplasia and nystagmus were present in all patients, with 83.3% (5/6 patients) showing evidence of intracranial misrouting. Prior to 2013, visual pathway developmental defects of foveal hypoplasia and intracranial misrouting were considered pathognomonic of albinism, and the associated defects in the melanin-biosynthesis pathway [4,6]. It has since been suggested that *SLC38A8*-related features occur in a melanin-independent manner with no effect on pigmentation. In our cohort two families were described as having paler skin (Family 2 and 5) and fair hair (Family 5), hence were clinically diagnosed with oculocutaneous/ocular albinism prior to genetic testing. Over time, the affected individuals in both families reached similar levels of pigmentation to their parents and siblings. In all the other reported families in the literature, there were no signs of cutaneous hypopigmentation. In addition, more variability in grade of foveal hypoplasia is seen in patients with albinism and *PAX6* variants compared to *SLC38A8* patients, who have a more severe retinal phenotype indicating an earlier arrest in foveal development [12].

Anterior segment dysgenesis (ASD) encompasses a spectrum of clinical features originating from maldevelopment of the anterior segment, which can involve all or any part of the cornea, anterior chamber, trabecular meshwork, iris, ciliary body, and lens. Our cohort, together with published cases report ASD features ranging from posterior embryotoxon alone to Axenfeld anomaly, blue-dot cataract and shallow anterior chambers. The prevalence of posterior embryotoxon in the younger (≤20 years of age) population has been reported to be 22.5% [17]. Analysis of all *SLC38A8* reported cases reveals a similar prevalence of 22% (13/58), but in our cohort alone, this increases to 33% (3/9). The *SLC38A8* variants in all patients with ASD were not localised to the same region/domain, so there was no genotype-phenotype correlation. Hence, it remains unclear if this is a variable feature of the disorder or a coincidental finding. Human gene expression datasets show that *SLC38A8* transcript (ENST00000299709.7) is differentially expressed across the eye including the foetal retina (7.75 log2(TPM + 1)), foetal RPE (5.56 log2(TPM + 1)), cornea endothelium (1.03 log2(TPM + 1)) and stem cell derived lens (2.92 log2(TPM + 1)) [18]. This pan-ocular expression is similar to *PAX6*, which also plays a significant role in foveal and anterior segment development; hence, further deeper phenotyping may be required with the use of anterior segment OCT to carefully capture the morphology of these affected eyes.

Isolated foveal hypoplasia without optic nerve misrouting or anterior segment dysgenesis was found in one family (family 1). Differential diagnoses includes autosomal dominant foveal hypoplasia-1 (FVH1, OMIM #136520), also referred to as foveal hypoplasia-1 with or without anterior segment anomalies and/or cataract, which is caused by heterozygous missense variants in the *PAX6* gene on chromosome 11p13 [19,20]. Hemizygous mutations in *GPR143* on chromosome Xp22, commonly cause ocular albinism type I (OMIM 300500); however, in a smaller number of cases, it has been reported to cause isolated congenital nystagmus-6 (OMIM 300814). In a six-generation Chinese family with a c.266C>T, p.(Ser89Phe) variant, affected members displayed nystagmus and foveal hypoplasia, without any typical signs of ocular albinism such as iris transillumination defects, fundus hypopigmentation and intracranial misrouting [21]. In a Korean family with a *GPR143* c.623C>A, p.(Ala208Glu) variant, one affected male displayed typical ocular albinism signs with severe hypopigmentation with clearly visible choroid vessels in the entire retina and eye movement recording showed a horizontal, conjugate pendular nystagmus. His affected sibling had isolated foveal hypoplasia with no nystagmus nor iris or fundus hypopigmentation [22]. Electrophysiology was not reported in this study. Care should be taken as iris and fundus hypopigmentation may not always be apparent in Asians. 

All but one of the patients in this study were solved through whole genome sequencing facilitated by the 100,000 Genomes Project, highlighting its utility to determine a molecular diagnosis, especially where the phenotypic overlap with albinism, aniridia and other ocular conditions can lead to potential misdiagnosis. Recognising the phenotype of *SLC38A8*-oculopathy (and pattern of inheritance) will enable the clinical genetics team to apply supporting evidence for pathogenicity (PP4) when required to confirm a molecular diagnosis of a *SLC38A8* variant of uncertain significance [23]. In addition, reaching an accurate diagnosis is especially important for informed genetic counselling and family planning advice, given the different inheritance patterns related to the other disease phenocopies. There are emerging gene-based applications for treating albinism [24] such as adeno-associated virus (AAV)-based Oa1 gene therapy which has been demonstrated in knockout mice models for ocular albinism [25] and with Tyr oculocutaneous albinism type 1 [26]. Successful CRISPR/Cas9 gene editing of *Tyr* has also been demonstrated for albinism in rabbits [27]. These pigment targeted gene-based therapies will be relevant for future therapies of patients with albinism, for example, through the restoration of tyrosinase production, and will depend on accurate genetic diagnosis.

*SLC38A8*-oculopathy may well be under diagnosed because of its subtle presentation, easily confused with albinism. Current molecular diagnostic rates for albinism and nystagmus using WGS are approximately 28% [13]. Until recently, *SLC38A8* was not included in the albinism and nystagmus targeted gene panels. As found with patients presenting with nystagmus [28], more comprehensive gene panels offered as an initial assessment will limit such diagnostic delays. Detailed phenotyping of patients will also help to characterise the pan-ocular features associated with this condition. 

## 4. Materials and Methods

### 4.1. Patients and Genetic Analysis

All patients attended the ocular genetics service at MEH. Patients and relatives gave written informed consent for participation in this study through either the Genetic Study of Inherited Eye Disease (REC reference 12/LO/0141) or Genomics England 100,000 Genomes project (REC reference 14/EE/1112). Families 1-6 comprising of probands and one or two unaffected parents where available) had undergone WGS analysis through participation in the 100,000 Genomes Project [29]. Detailed by Taylor et al. [30], genomic DNA was processed using an Illumina TruSeq DNA PCR-Free Sample Preparation kit (Illumina Inc., San Diego, CA, USA) and sequenced using an Illumina HiSeq X Ten high-throughput sequencing platform, generating minimum coverage of 15 X for >97% of the callable autosomal genome. Readings were aligned to either build GRCh37 or GRCH38 of the human genome using an Isaac aligner (Illumina Inc., San Diego, CA, USA). Single-nucleotide variants (SNVs) and indels (insertions or deletions) were identified using Platypus soft-ware (version 0.8.1; and annotated using Cellbase software (https://github.com/opencb/cellbase). Variant filtering was performed using minor allele frequency (MAF) < 0.001 in publicly available and in-house data sets, predicted protein effect, and familial segregation. Surviving variants were prioritized using the Retinal disorders version 2.120 (https://panelapp.genomicsengland.co.uk/panels/307/) and the Albinism or congenital nystagmus version 1.10 (https://panelapp.genomicsengland.co.uk/panels/511/) virtual gene panels. 

The proband from family 7 underwent targeted albinism and nystagmus gene panel testing through the Rare & Inherited Disease Genomic Laboratory at Great Ormond Street Hospital (London, UK). Mutation screening was carried out by next generation sequencing with library preparation using the Agilent focused clinical exome +1 kit followed by sequencing on the Illumina platforms. Data were analysed using an in-house pipeline and virtual gene panels including *SLC38A8*, with all mutations confirmed by Sanger sequencing. 

Likely pathogenicity of the novel missense variant was assessed using the predictive algorithms PolyPhen-2 [31], SIFT [32] and Mutation Taster [33].The genetic results were reviewed by a multidisciplinary team (including molecular biologists, clinical geneticists, as well as the ophthalmology specialist managing the family), to confirm variant pathogenicity, prevalence in publicly available genome databases, the clinical phenotype and mode of inheritance, before the molecular diagnosis was established. The datasets (variants) generated for this study were submitted to ClinVar (https://www.ncbi.nlm.nih.gov/clinvar/). We describe nine patients from seven unrelated families who were clinically diagnosed with bilateral foveal hypoplasia and nystagmus, and molecularly confirmed biallelic mutations in *SLC38A8.*

### 4.2. Clinical Assessment

Ophthalmic examination included full orthoptic assessment, refraction, best corrected visual acuity (VA) measured using LogMAR or Cardiff cards for preverbal children up to 36 months of age; slit lamp examination, and fundus examination were recorded with anterior segment and ultra-widefield (UWF) fundus colour imaging using the Haag-Streit slit lamp camera (Haag-Streit Holdings AG, Köniz, Switzerland) and Optos^®^ California (Optos plc), respectively. Investigations included spectral domain optical coherence tomography (SD-OCT), fundus autofluorescence, eye movement recordings (EMR) to assess nystagmus waveforms, electrophysiology to detect chiasmal misrouting of retinal ganglion cells through visual evoked potentials (VEP) against ISCEV standards, and either multichannel flash VEP alone (for younger paediatric patients) or a combination of both flash and pattern VEP. Due to the nystagmus and foveal hypoplasia seen in all patients, retinal imaging was challenging due to fixation difficulties and compliance in young children. Multiple line scan SD-OCT was attempted, but reverted to multiple single line scans where not possible to capture the foveal pit.

## Figures and Tables

**Table 1:** *SLC38A8* variants identified in our cohort using reference sequence NM_001080442.2. Abbreviations: gnomAD, genome aggregation database; TM, transmembrane; HOM, homozygous; HET, heterozygous.

Family ID	Ethnicity	Consanguinity	Variants	Exon	Consequence	Protein Domain	Zygosity	Reported	gnomAD
1 (26320)	South Asian Bangladesh	Yes	c.264C>G p.(Tyr88*)	2	Nonsense	Intracellular between TM domains 1 and 2	HOM	Novel	0.0002614 in South Asians only
2 (23089)	South Asian Indian	Yes	c.264C>G p.(Tyr88*)	2	Nonsense	Intracellular between TM domains 1 and 2	HOM	Novel	0.0002614 in South Asians only
3 (26350)	South Asian Pakistan	Yes	c.264C>G p.(Tyr88*)	2	Nonsense	Intracellular between TM domains 1 and 2	HOM	Novel	0.0002614 in South Asians only
4 (26364)	South Asian Sri-Lanka	Yes	c.698A>G p.Glu233Gly	6	Missense	TM6	HOM	Novel	Absent
5 (26237)	Caucasian British	No	c.435G>A p.Trp145*	3	Nonsense	Extracellular between TM domains 3 and 4	HET	Novel	Absent
			c.632+1G>A	IVS4	Splice donor	Extracellular between TM domains 5 and 6	HET	Novel	Absent
6 (16812)	Caucasian Spanish-British	No	c.923C>G p.Thr308Ser Del Ex7-8	7	Missense splice site impact Deletion	TM8 TM7-TM10	HET HET	[9] [9]	Absent Absent
7 (28327)	Caucasian Ashkenazi Jewish	No	c.848A>C p.(Asp283Ala)	7	Missense	Extracellular between TM domains 7 and 8	HOM	[8,9]	0.0002653 in gnomAD 0.006178 in AJs

**Table 2:** Clinical findings in patients with *SLC38A8* variants. Best corrected visual acuity (BCVA) recorded in logMAR (OD, right eye, and OS, left eye).

Family ID/Patient	Ethnicity	Age (Years)	Gender	BCVA OD	BCVA OS	Refraction	Strabismus	Nystagmus	Anterior Segment	Foveal Hypoplasia	Electrodiagnostic Testing
1-1	South Asian Bangladesh	3	Male	Objects to occlusion	Objects to occlusion	R: +3.50/−2.50 × 180 L: +3.50/−3.50 × 180	Left Exotropia	Pendular horizontal	Normal	Present	No evidence of chiasmal misrouting
2-1	South Asian Indian	32	Female	0.6	0.6	R: −1.75/−3.50 × 180 L: −7.00/−2.50 × 170	Left Esotropia	Pendular horizontal	Normal	Grade 4	Evidence of chiasmal misrouting
2-2		16	Male	0.82	0.78	R: −0.75/−3.50 × 10 L: +1.50/−4.00 × 175	Right Exotropia	Pendular horizontal	Posterior embryotoxon	Grade 4	Evidence of chiasmal misrouting
3-1	South Asian Pakistan	21	Female	0.7	0.9	R: +0.75/−2.50 × 30 L: +0.75/−2.50 × 75	Left Exotropia	Pendular horizontal	Normal	Present	Not undertaken
4-1	South Asian Sri-Lanka	39	Female	0.6	0.6	Not available	Left Exotropia	Pendular horizontal	Bilateral Posterior embryotoxon Bilateral peripheral iris adhesions to the cornea Bilateral blue dot cataract	Grade 4	Evidence of chiasmal misrouting
5-1	Caucasian British	5	Female	0.6	0.6	R: +3.50DS L: +3.50DS	No deviation	Jerk with extended foveation	Normal	Grade 4	Evidence of chiasmal misrouting
5-2		2	Male	Objects to occlusion	Objects to occlusion	R: +5.00/−0.75 × 180 L: +5.00/−0.75 × 180	No deviation	Pendular horizontal	Normal	Present	Not undertaken
6-1	Caucasian Spanish-British	36	Female	0.9	1	Not available	Left Esotropia	Rotary	Bilateral shallow anterior chamber	Grade 4	Evidence of chiasmal misrouting
7-1	Caucasian Ashkenazi Jewish	16	Female	0.42	0.4	R: +2.50/−1.50 × 165 L: +1.50/−1.50 × 180	Right Exotropia	Pendular horizontal	Peripheral iris transillumination	Grade 4	Evidence of chiasmal misrouting

## Data Availability

The variants reported in this paper have been deposited into the ClinVar database (https://www.ncbi.nlm.nih.gov/clinvar/) at the National Center for Biotechnology Information under accession numbers RCV001270481, SCV001450776, SCV001450777 and SCV001450778.

## Supplementary Materials

The following are available online at https://www.mdpi.com/1422-0067/22/3/1130/s1, Table S1: Published variants in *SLC38A8*, Table S2: Percentage of phenotypic features seen in previously documented cases.
